# Investigating the Tocopherol Contents of Walnut Seed Oils Produced in Different European Countries Analyzed by HPLC-UV: A Comparative Study on the Basis of Geographical Origin

**DOI:** 10.3390/foods11223719

**Published:** 2022-11-19

**Authors:** Petros D. Mitsikaris, Lambros Kokokiris, Agathi Pritsa, Athanasios N. Papadopoulos, Natasa P. Kalogiouri

**Affiliations:** 1 Laboratory of Chemical Biology, Department of Nutritional Sciences and Dietetics, International Hellenic University, Sindos, 57400 Thessaloniki, Greece; 2 Laboratory of Analytical Chemistry, Department of Chemistry, Aristotle University of Thessaloniki, 54124 Thessaloniki, Greece

**Keywords:** walnut, seed oil, tocopherols, HPLC-UV, geographical origin

## Abstract

A rapid HPLC-UV method was developed for the determination of tocopherols in walnut seed oils. The method was validated and the LODs ranged between 0.15 and 0.30 mg/kg, while the LOQs were calculated over the range of 0.50 to 1.00 mg/kg. The accuracy values ranged between 90.8 and 97.1% for the within-day assay (*n* = 6) and between 90.4 and 95.8% for the between-day assay (*n* = 3 × 3), respectively. The precision of the method was evaluated and the RSD% values were lower than 6.1 and 8.2, respectively. Overall, 40 samples of walnuts available on the Greek market, originating from four different European countries (Greece, Ukraine, France, and Bulgaria), were processed into oils and analyzed. One-way ANOVA was implemented in order to investigate potential statistically significant disparities between the concentrations of tocopherols in the walnut oils on the basis of the geographical origin, and Tukey’s post hoc test was also performed to examine exactly which varieties differed. The statistical analysis of the results demonstrated that the Ukrainian walnut seed oils exhibited significantly higher total concentrations compared to the rest of the samples.

## 1. Introduction

Walnuts (*Juglans regia* L.) are a prominent member of the culinary nuts family. Their chemical composition, both macronutrient- and micronutrient-wise [1,2], suggests that they are a useful choice in a healthy and balanced diet. According to the FAO [3], 3,323,964 tons of walnuts were produced worldwide in 2020, while China, USA, Iran, Turkey, and Mexico were the top producers. The bioactive compounds in walnuts and walnuts oils have been linked with several beneficial effects to human health, such as anti-inflammatory [4], cardioprotective [5], and anti-proliferative [6] effects, among others. Furthermore, walnut consumption also aids in slowing down degenerative brain diseases [7,8] and has also been associated with hunger suppression [9], rendering it an effective tool in a weight-loss plan.

Tocopherols (α-, β-, γ-, and δ-) are part of the commonly known vitamin E complex, along with the corresponding tocotrienols. Their molecular structure consists of a chromanol ring and a saturated phytyl side chain located at the C2 position. Within the human organism they act as powerful antioxidants, as has been proven by both in vivo [10] and in vitro [11] studies. Specifically, their main function is to protect against the non-enzymatic peroxidation of polyunsaturated fatty acids that construct the bilayer cellular membrane [12]. Tocopherols are plant-derived [13], meaning they have to be provided to humans through diet or supplementation. One of their main characteristics is that they are lipid-soluble, and this is the reason why they can only be found in high-fat plant foods [14,15], such as walnuts.

The exploration of the tocopherol content in walnut oil samples is a broadly analyzed topic in the literature. There are an array of studies available discussing walnut oil’s tocopherol content on the basis of the variety, maturity level, crop year, harvest time, and storage conditions [16,17,18,19,20,21,22,23,24,25,26,27]. However, there is no information available regarding the walnut oil tocopherol profile on the basis of the geographical origin. This is a gap that has to be filled, taking into consideration that domestic and imported products are both widely available in the Western world and consumers are concerned about which to select.

Several analytical protocols have been proposed in the literature for tocopherol analysis, employing high-pressure liquid chromatography coupled to diode array (HPLC-DAD) [27,28,29], UV–Vis [30,31], fluorescence (FLD) [32], or mass spectrometric (MS) detectors [33,34,35,36]. Undoubtedly, UV detectors allow a fast, reliable, and cost-effective analysis, especially when it comes to such a small number of compounds, where separation and identification can be achieved without much adversity. The flagship of the analytical workflow techniques, however, is sample preparation. Several prolonged and laborious protocols have been suggested, including some that involve the use of substantial volumes of organic solvents in a Soxhlet apparatus [37]. Solid liquid extraction (SLE) is also frequently used, but in most cases requires the use of large amounts of solvents [37,38]. The use of solid-phase extraction (SPE) allows for a reduction in the amount of toxic solvents but demands the fulfillment of various steps throughout the sample preparation process, as well as the acquisition of cartridges [27]. The objective is to choose the right approach in order to determine the bioactive analytes that are present in the samples, eliminate the use of time-consuming extraction steps, and minimize the use of organic solvents.

The next step after the chromatographic analysis involves data mining. The statistical analysis of the experimental data enables the interpretation of the results. Statistical and chemometric tools are widely used in authenticity studies to support the outcomes that are acquired from the experimental results [30,31,39,40].

The goal of this study is to propose a rapid HPLC-UV analytical protocol that could be applied in the investigation of the authenticity of walnut seed oils prepared from walnut seeds that are available in the Greek market, originating from four European countries, namely Greece, Ukraine, France, and Bulgaria. The concentrations of tocopherols among the analyzed samples of different geographical origins were examined using a one-way ANOVA. Tukey’s post-hoc test was also performed to examine exactly which samples from different geographical origins differed from each other.

## 2. Materials and Methods

### 2.1. Chemicals and Reagents

The HPLC-grade acetonitrile (ACN) and HPLC-grade methanol (MeOH) were acquired from Carl-Roth (Carlsruhe, Germany), while the 2-propanol (IPA) and heptane were purchased from Panreac-AppliChem (Darmstadt, Germany). The ultrapure water was provided by a Milli-Q water purification system (Millipore, Bedford, MA, USA). The standard compounds that were utilized were all purchased by Sigma-Aldrich (Steinheim, Germany), namely α-tocopherol (96%), β-tocopherol (96%), γ-tocopherol (96%), and δ-tocopherol (96%). For each one of the standard compounds, stock solutions at a concentration of 1000 μg/mL were prepared in MeOH and they were then stored in dark brown bottles at −20 °C. 

### 2.2. Walnut Samples

Forty samples of dried walnut kernels (500 g each) were used in the study, and they were all available on the Greek market. Specifically, 10 samples that originated from various regions of Macedonia and Thessaly in Greece, 10 samples of walnuts produced in Bulgaria, 10 samples produced in Ukraine, and 10 samples produced in France were provided by traders. All samples were acquired in 2021. The walnut kernels were separated from their shells and subsequently chopped and homogenized in a mixer. All samples were stored at −20 °C until further processing and analysis.

### 2.3. Instrumentation

The chromatographic analysis was performed using an Agilent 1220 Infinity HPLC-UV system (Agilent Technologies, Santa Clara, CA, USA). The system comprised of: (i) the degasser, (ii) the column oven, (iii) the manual injector, and (iv) the UV detector. The OpenLAB software (Agilent Technologies, Santa Clara, CA, USA) and the Method and Run control package were employed to monitor the analysis. The sonication of the samples was performed in a MRC:DC-150-H ultrasonic bath provided by MRC (Essex, UK). A vortex mixer purchased from VELP Scientifica (Usmate Velate, Italy) was used for agitation. Centrifugation was carried out using a 3-16PK centrifuge system supplied by Sigma (Osterode am Harz, Germany). Prior to the injection in the chromatographic system, all samples were filtered through QMax RR 25 mm 0.22 μm PTFE syringe filters purchased from Frisenette ApS (Knebel, Germany). 

### 2.4. Chromatographic Analysis

For the separation of the analytes, a reversed-phase (RP) Kromasil C18 (4.6 × 250 mm, 5 μm) analytical column was used, provided by Macherey-Nagel (Dueren, Germany). A binary gradient elution program consisting of MeOH (A) and ACN (B) was used for the separation of tocopherols. The temperature in the column oven was maintained at 28 °C. The elution program lasted for 15 min in total. The gradient program started with 50% A and remained stable for seven min, then gradually increased to 100% A until the 12 min mark, and remained stable for the last 3 min [31]. The flow rate was set to 1 mL/min and the absorbance was measured at 295 nm.

### 2.5. Sample Preparation

For sample preparation, a slightly altered version of the extraction procedure previously introduced by Martakos et al. [41] was employed. First of all, the walnut seeds were thoroughly homogenized in a porcelain mortar. After, 1 g of solid sample was weighed in a falcon tube and 10 mL of heptane was added. The solution was vigorously agitated in a vortex mixer for 1 min and was then placed in an ultrasonic bath at 40 °C for 20 min. The samples were subsequently centrifuged at 8000 rpm for 10 min. In a following step, the organic layer was transferred into the rotary evaporator and evaporated under vacuum to obtain the pure oil. Then, 100 mg of oil was weighed and extracted with 400 μL of isopropanol. The mixture was vortexed during 1 min, centrifuged at 8000 rpm for 10 min, and finally, the aliquot was collected and filtered through 0.22 μm PTFE syringe filters prior to the injection in the chromatographic system.

### 2.6. Method Validation

Linearity, accuracy, precision, limits of detection (LODs), and limits of quantification (LOQs) were assessed to validate the method. Linearity studies were conducted at the concentration range LOQ—50 mg/kg, by plotting the peak area versus the concentration of the standard compounds for 7 calibration points. The r^2^ values of the standard calibration curves were calculated in order to assess the linearity of the method. The LOQs were considered to be the lower point of the calibration curve that corresponded to a signal-to-noise ratio (S/N) higher than 10. In order to calculate the LOD values, the LOQ of each analyte was divided by 3.3 [42]. Accuracy and precision were evaluated using a pool sample spiked at three different concentration levels (0.5, 25 and 50 mg/kg). The relative recoveries (%R) were calculated by means of recovery percentage, by comparing the found and added concentrations of the examined analytes, expressing accuracy. The relative standard deviations (%RSDs) were calculated to assess the precision of the method. Repeatability was assessed by measuring within-day precision using six replicates (*n* = 6), and reproducibility was evaluated by performing triplicate analysis of a pool sample spiked at three different concentrations (0.5, 25 and 50 mg/kg) within three consecutive days. 

### 2.7. Statistical Analysis

The statistical analysis was performed using IBM SPSS Statistics 21. As the assumption of normality was not met by tocopherol concentration values in some groups, the concentrations of each tocopherol were compared between groups using the Kruskal–Wallis non parametric test at α = 0.05. One-way ANOVA was performed to explore potential significant disparities between the samples, both in the concentration of every single tocopherol and in their total sum, as well. The confidence level was set at α = 0.05. To discover this, a post hoc analysis was carried out. There isa large variety of post hoc tests available, all of which have their own pros and cons [43]. In this study, we chose to perform Tukey’s test, which is the most commonly used one [44]. The MetaboAnalyst 5.0 package was used to create box plots and present the concentrations of the determined tocopherols in the analyzed samples [45].

## 3. Results

### 3.1. Method Validation Results

Table 1 presents the analytical parameters of the HPLC-UV method. The LODs were found to range between 0.15 to 0.30 mg/kg, and the LOQ ranged between 0.50 to 1.00 mg/kg. The method precision was good since the %RSD values of the within-day (*n* = 6) and between-day assays (*n* = 3 × 3) were lower than 6.1 and 8.2, respectively. The accuracy was assessed by means of relative percentage of recovery (%R) were calculated at three concentration levels (0.5, 25, 50 mg/kg), and ranged between 90.8 and 97.1% for the within-day assay (*n* = 6) (Table 2), and between 90.4 and 95.8% for between-day assay (*n* = 3 × 3) (Table 3).

### 3.2. Walnut Seed Oil Analysis

**Figure 1:**
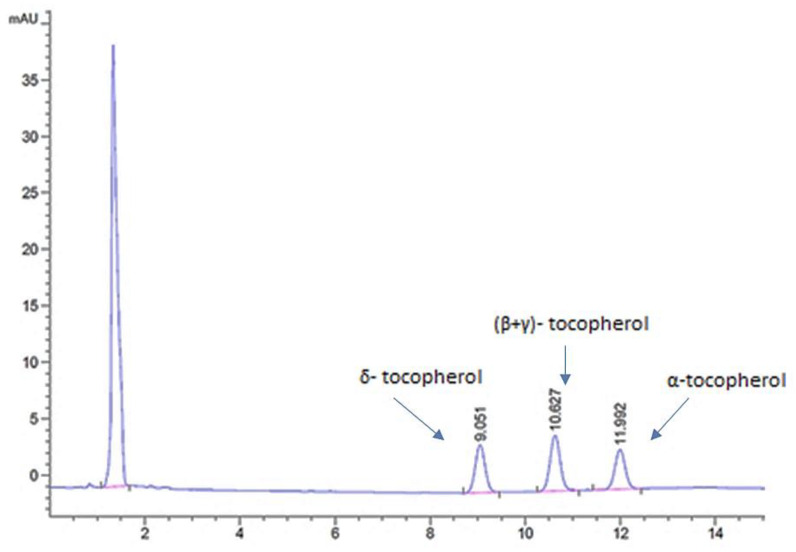
Characteristic chromatogram of a standard mixture of tocopherols (α-tocopherol: 5 mg/kg; (β+γ)-tocopherol: 5 mg/kg; δ-tocopherol: 5 mg/kg) monitored at 295 nm.

Overall, 40 samples originating from 4 different countries were analyzed in triplicate, and the contentrations of α-tocopherol, (β+γ)-tocopherol, and δ-tocopherol were determined. The separation of the tocopherols was accomplished within twelve minutes, as it is shown in the characteristic chromatogram of a standard mixture of tocopherols (α-tocopherol: 5 mg/kg; (β+γ)-tocopherol: 5 mg/kg; δ-tocopherol: 5 mg/kg) monitored at 295 nm, presented in Figure 1. The β- and γ-tocopherol are isomers and were quantified as a sum [46]. Table 4 presents the determined analytes along with the molecular formulas, molecular structures, and retention times (RTs). The β- and γ-tocopherol are isomers, and subsequently they eluted at the same RT [27,30,31]. 

### 3.3. Quantitative Analysis of Tocopherols

The concentration ranges (minimum and maximum values) of the tocopherols expressed in mg per kg of walnut seed oil, are presented in Table 5. The average concentration ranges and their mean values (±SD), as well as their average total concentrations are presented in Table 6. The sum of β- and γ-tocopherol was proven to be the most abundant in all samples, owing to the high concentrations of γ-tocopherol in the walnuts [16]. The second most abundant tocopherol was δ-tocopherol, whileα-tocopherol was the least abundant. 

**Figure 2:**
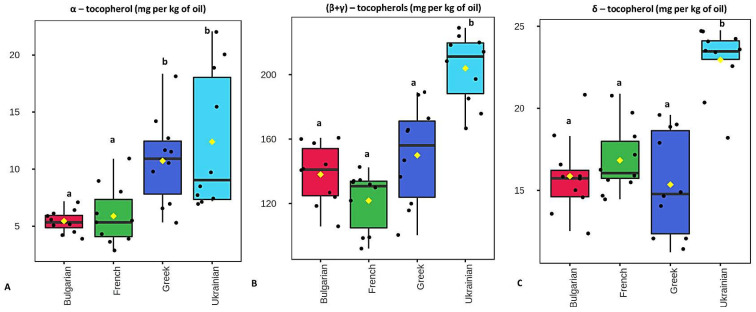
Box plot of α-tocopherol (**A**) and the sum of β- and γ-tocopherols (**B**) and δ-tocopherol in oils (**C**) from Bulgarian, French, Greek, and Ukrainian walnuts. Box plot values highlighted with different Latin letters differ significantly at α = 0.001.

**Figure 3:**
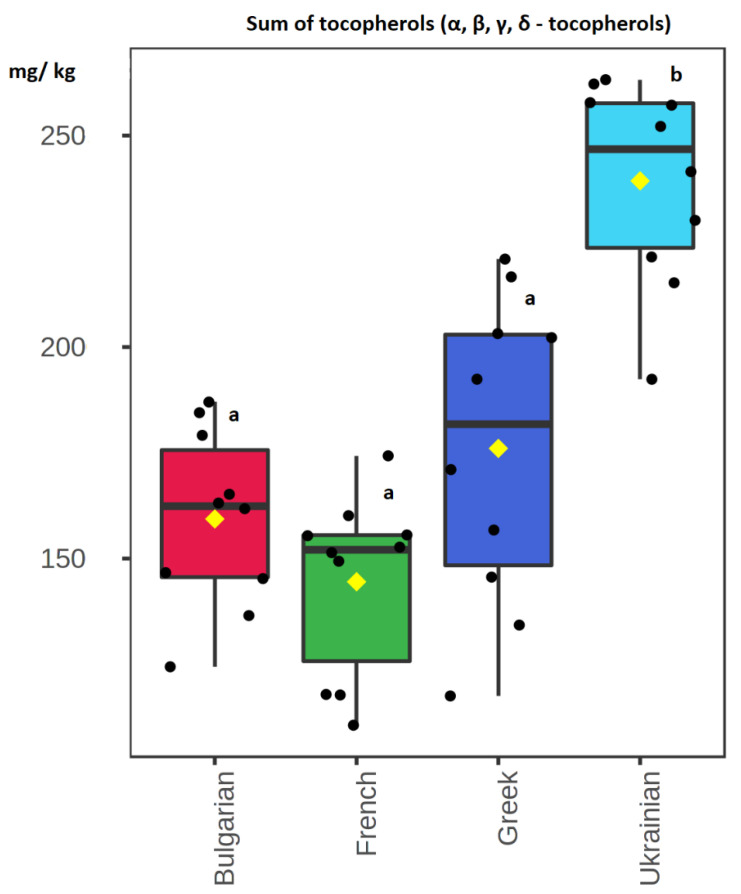
Box plot of total tocopherol contents (sum of α-, β-, γ- and δ-tocopherols) in oils from Bulgarian, French, Greek, and Ukrainian walnuts. Box plot values highlighted with different Latin letters differ significantly at α = 0.001.

Box and whisker plots were created for each compound to graphically depict their concentrations among the different European regions. The concentration levels of α-tocopherol did not differ significantly between Greek and Ukrainian walnuts. However, these concentration levels were significantly higher compared to those determined in Bulgarian and French walnuts (x2 (3) = 19.9, *p* < 0.001, Figure 2A). The sum of (β and γ)-tocopherols and the concentration levels of δ- tocopherol were determined in significantly higher levels in Ukrainian walnuts (x2 (3) = 23.6, *p* < 0.001 for sum of β- and γ-tocopherols, Figure 2B; x2 (3) = 20.6, *p* < 0.001, for δ tocopherol, Figure 2C). Similarly, the average total concentrations of tocopherols were found to be significantly higher in Ukrainian walnuts (x2 (3) = 22.9, *p* < 0.001, Figure 3). δ-Tocopherol was found to be the most abundant tocopherol in Ukrainian walnuts with a mean concentration equal to 23 ± 2 mg per kg of oil. The second most abundant concentration was determined in French samples (17 ± 2 mg/kg). The Bulgarian and Greek walnuts mean values were 16 ± 2 mg/kg and 15 ± 3 mg/kg, respectively. The total tocopherol content was highest in Ukrainian samples, presenting a mean value equal to 239 ± 23 mg per kg of oil. The Greek walnuts had the second highest mean value of 176 ± 34 mg/kg. The Bulgarian walnuts mean concentration was 159 ± 20 mg/kg, while the French walnuts demonstrated a mean value of 145 ± 20 mg/kg.

The concentration ranges reported were in accordance with studies published in the literature. There is a wide variety of studies available that confirm that γ-tocopherol is of the highest concentration in walnut seed oils, no matter the cultivar or the geographical origin of the sample [19,20,22,23,24,25,47,48,49,50].

Concerning α-tocopherol, in particular, some of the aforementioned studies have reported higher values compared to those reported in this study, at up to 45 mg per kg of walnut oil [20,21,24]. The range of concentrations is even wider for the sum contents of β- and γ-tocopherols, since some studies have reported the sum of β- and γ-tocopherols at the lowest limit of 35 mg per kg of oil, which is significantly lower compared to the concentrations found in this study [20]. On the other hand, higher concentrations have been reported in crops originating from Argentina and Turkey, supporting the idea that there is a correlation between the tocopherol content and the geographical origin [18,24]. As for δ-tocopherol, the concentration ranges reported in the literature are similar to those determined in the present study. 

## 4. Conclusions

A rapid protocol was developed to determine the concentrations of tocopherol homologues in walnut samples originating from four different European regions (Greece, Ukraine, France, and Bulgaria) using HPLC-UV. According to the results, the highest concentrations were determined in Ukrainian walnuts, the second largest were found in Greek walnuts, and then the Bulgarian and the French walnuts followed. The results were analyzed with one-factor ANOVA, and it was revealed that the samples differed significantly from each other (*p*-value < 0.001), indicating that big discrepancies do exist on the basis of the samples’ geographical origin. Furthermore, the Ukrainian walnut seed oils presented a significantly higher concentration compared to the rest of the samples. Finally, the Greek walnuts possessed a significantly higher mean concentration of α-tocopherol compared to the French and Bulgarian walnuts and a significantly higher mean concentration of β- plus γ-tocopherols compared to the French walnuts. 

## Figures and Tables

**Table 1:** HPLC-UV analytical parameters.

Compound	Calibrationequation	Linear Range (mg/kg)	r^2^	LOD (mg/kg)	LOQ (mg/kg)
α-tocopherol	y = 5.68x − 1.23	1–50	0.999	0.30	1.00
β+γ-tocopherol	y = 6.94x + 0.19	0.5–50	0.997	0.15	0.50
δ-tocopherol	y = 5.89x + 0.33	0.8–50	0.996	0.27	0.80

LOD: limit of detection; LOQ: limit of quantitation.

**Table 2:** Recovery (%R) rates for the evaluation of the repeatability.

Compound	Low Concentration (%R, *n* = 6)	%RSD	Medium Concentration (%R, *n* = 6)	%RSD	High Concentration (%R, *n* = 6)	%RSD
α-tocopherol	92.2	5.4	90.8	5.8	96.5	6.1
β+γ-tocopherol	94.1	3.6	92.5	2.9	97.1	5.8
δ-tocopherol	93.5	6.5	96.9	4.3	93.4	5.3

**Table 3:** Recovery (%R) rates for the evaluation of the reproducibility.

Compound	Low Concentration (%R, *n* = 3 × 3)	%RSD	Medium Concentration (%R, *n* = 3 × 3)	%RSD	High Concentration (%R, *n* = 3 × 3)	%RSD
α-tocopherol	93.7	6.5	93.3	6.5	95.5	8.2
(β+γ)-tocopherol	94.3	7.3	94.1	8.3	91.2	6.9
δ-tocopherol	95.8	5.8	90.4	7.5	92.4	7.5

**Table 4:** Molecular formulas, molecular structures, and retention times.

Compound	Molecular Formula	Molecular Structure	RT (min)
α-tocopherol	C_29_H_50_O_2_	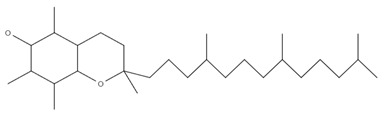	12.0
β-tocopherol	C_28_H_48_O_2_	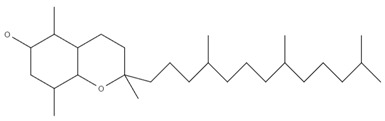	10.6
γ-tocopherol	C_28_H_48_O_2_	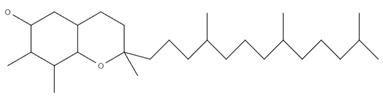	10.6
δ-tocopherol	C_27_H_46_O_2_	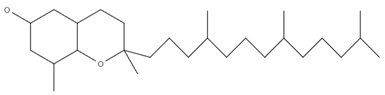	9.1

RT: retention time.

**Table 5:** Concentration ranges (mg per kg of walnut seed oil) of the samples.

Tocopherol	Greek Walnuts	French Walnuts	Ukrainian Walnuts	Bulgarian Walnuts
α-	5.3–18.4	2.9–10.9	7.2–22.1	4.1–7.2
β- and γ-	100.3–189	92.0–142.5	166.9–229.0	105.9–160.8
δ-	11.2–19.6	14.5–20.9	18.3–24.8	12.5–20.9
Total	117.5–220.8	110.6–174.3	192.4–263.2	124.4–187.0

**Table 6:** Mean values ± SD (mg per kg of walnut seed oil) of samples.

Tocopherol	Greek Walnuts (*n* = 10)	French Walnuts (*n* = 10)	Ukrainian Walnuts (*n* = 10)	Bulgarian Walnuts (*n* = 10)
α-	10± 4	6 ± 3	12 ± 6	5.5 ± 1.0
β- and γ-	150 ± 30	122 ± 17	204 ± 21	138.0 ± 17.8
δ-	15± 3	17 ± 2	23 ± 2	16 ± 2
Total	176 ± 34	145 ± 20	239± 23	159 ± 20
